# Maternal provisioning of an obligate symbiont in a sponge

**DOI:** 10.1002/ece3.10012

**Published:** 2023-05-02

**Authors:** Tyler J. Carrier, Lara Schmittmann, Sabrina Jung, Lucía Pita, Ute Hentschel

**Affiliations:** ^1^ GEOMAR Helmholtz Center for Ocean Research Kiel Germany; ^2^ Zoological Institute, Christian‐Albrechts University of Kiel Kiel Germany; ^3^ Department Marine Biology and Oceanography Institute of Marine Sciences (ICM‐CSIC) Barcelona Spain

**Keywords:** animal–microbe, development, life history evolution, marine invertebrate, trade‐off

## Abstract

The transmission of microbes from mother to offspring is an ancient, advantageous, and widespread feature of metazoan life history. Despite this, little is known about the quantitative strategies taken to maintain symbioses across generations. The quantity of maternal microbes that is provided to each offspring through vertical transmission could theoretically be stochastic (no trend), consistent (an optimal range is allocated), or provisioned (a trade‐off with fecundity). Examples currently come from animals that release free‐living eggs (oviparous) and suggest that offspring are provided a consistent quantity of symbionts. The quantity of maternal microbes that is vertically transmitted in other major reproductive strategies has yet to be assessed. We used the brooding (viviparous) sponge *Halichondria panicea* to test whether offspring receive quantitatively similar numbers of maternal microbes. We observed that *H*. *panicea* has a maternal pool of the obligate symbiont *Candidatus* Halichondribacter symbioticus and that this maternal pool is provisioned proportionally to reproductive output and allometrically by offspring size. This pattern was not observed for the total bacterial community. Experimental perturbation by antibiotics could not reduce the abundance of *Ca.* H. symbioticus in larvae, while the total bacterial community could be reduced without affecting the ability of larvae to undergo metamorphosis. A trade‐off between offspring size and number is, by definition, maternal provisioning and parallel differences in *Ca.* H. symbioticus abundance would suggest that this obligate symbiont is also provisioned.

## Graphical abstract

Transmission of microbes from mother to offspring is a widespread feature of metazoan life‐history and, despite this, little is known about which quantitative strategies are taken to maintain symbioses across generations. Here, we observed that the sponge *Halichondria panicea* has a maternal pool of an obligate symbiont that is provisioned proportionally to reproductive output and allometrically by offspring size. A trade‐off between offspring size and number is, by definition, maternal provisioning and parallel differences in the abundance of this obligate symbiont would suggest that this symbiont is also provisioned.

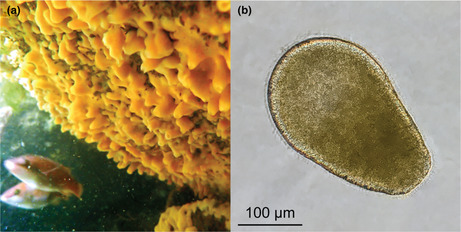

## 1 INTRODUCTION

Animals use their reproductive machinery to maintain symbioses across generations. The vertical transmission of maternal microbes is ancient, evolutionarily advantageous, and occurs for single mutualists as well as diverse prokaryotic communities (Bright & Bulgheresi, 2010; Carrier & Bosch, 2022; Funkhouser & Bordenstein, 2013; McFall‐Ngai, 2002; Moran & Wernegreen, 2000). Most major metazoan lineages have been observed to transmit microbes from mother to offspring [annelids (Davidson & Stahl, 2008), arthropods (Ferree et al., 2005), chordates (Hirose, 2015), cnidarians (Apprill et al., 2009), echinoderms (Carrier et al., 2021), flatworms (Jäckle et al., 2019), molluscs (Cary & Giovannoni, 1993), nematodes (Stevens et al., 2001), and sponges (Carrier et al., 2022; Díez‐Vives et al., 2022)]. These developmental symbionts have deep evolutionary origins and play integral roles in host biology and ecology, including acclimation (Foutaine et al., 2022), gametogenesis (Dedeine et al., 2001), and metamorphosis (Song et al., 2021).

**FIGURE 1:**
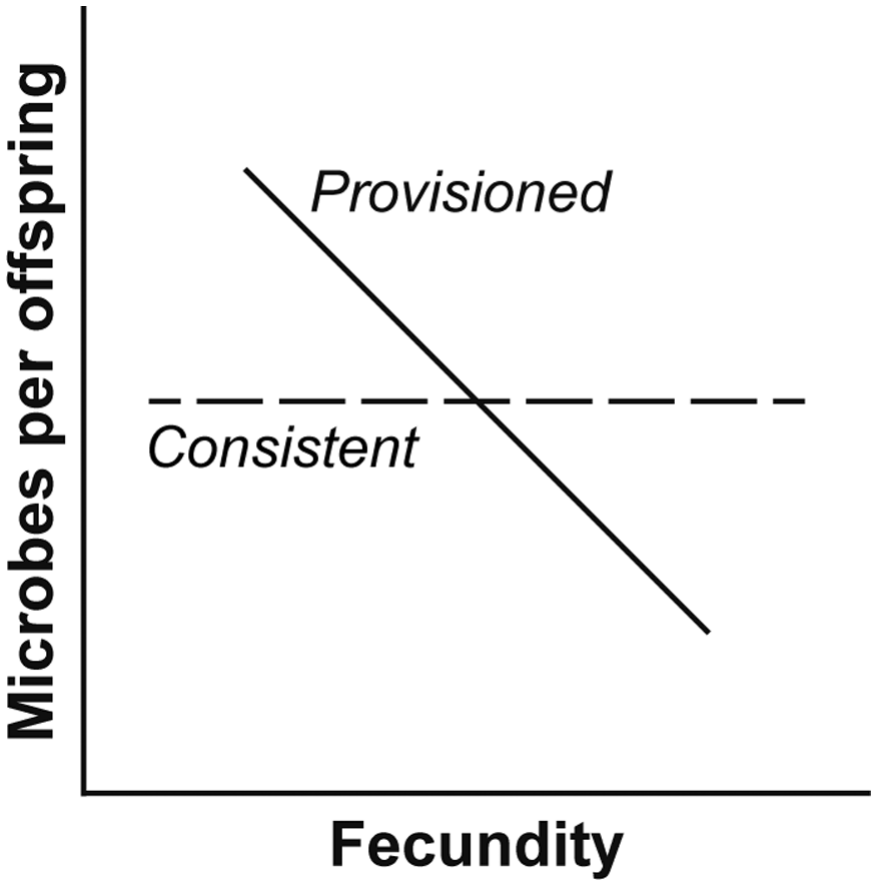
Theoretical strategies for quantitatively transmitting microbes to offspring. The number of maternal microbes that are provided to each offspring may be stochastic (no trend; not displayed), consistent (an optimal range is allocated; dashed line), or provisioned (a trade‐off with fecundity; solid line).

Despite being a widespread feature of metazoan life history, little attention has been given to understanding the quantitative strategies taken to transmit maternal microbes to the next generation. Each offspring could theoretically be provided a stochastic (no trend), consistent (an optimal range is allocated), or provisioned (a trade‐off with fecundity) quantity of maternal microbes (Figure 1). Examples in the literature suggest that offspring are provided a consistent quantity of maternal symbionts (Chamberland et al., 2017; Flórez & Kaltenpoth, 2017; Mira & Moran, 2002; Pons et al., 2022; Russell et al., 2018). The aphid *Uroleucon ambrosiae*, for example, provides ~10^3^ (± ~ 5%) cells of the nutritional mutualist *Buchnera* spp. to each egg, while the stinkbug *Megacopta punctatissima* produces one capsule with ~10^8^ cells of the obligate symbiont *Candidatus* Ishikawaella capsulate for every three to four eggs (Hosokawa et al., 2007; Mira & Moran, 2002).

Support for the vertical transmission of a consistent number of maternal symbionts stems from species that release free‐living eggs (i.e., are oviparous). Other major reproductive strategies have yet to be assessed. One strategy where vertical transmission is common and where we lack a quantitative understanding of these maternal microbes is viviparity (Bright & Bulgheresi, 2010; Carrier & Bosch, 2022). In this, fertilization occurs internally, early embryos are brooded within the maternal body, and late‐stage offspring are released into the environment. Viviparity is widespread among animals and is particularly common in marine sponges (Díez‐Vives et al., 2022). Brooding provides the adult sponge with a developmental window to transmit maternal microbes that are within the mesohyl (Björk et al., 2019; Carrier et al., 2022; Díez‐Vives et al., 2022; Maldonado, 2007; Maldonado & Riesgo, 2009; Riesgo et al., 2007). This is particularly evident in ‘low microbial abundance’ sponges, which inherit a select few dominant symbionts that are essential for maternal fitness (Carrier et al., 2022; Song et al., 2021).

**FIGURE 2:**
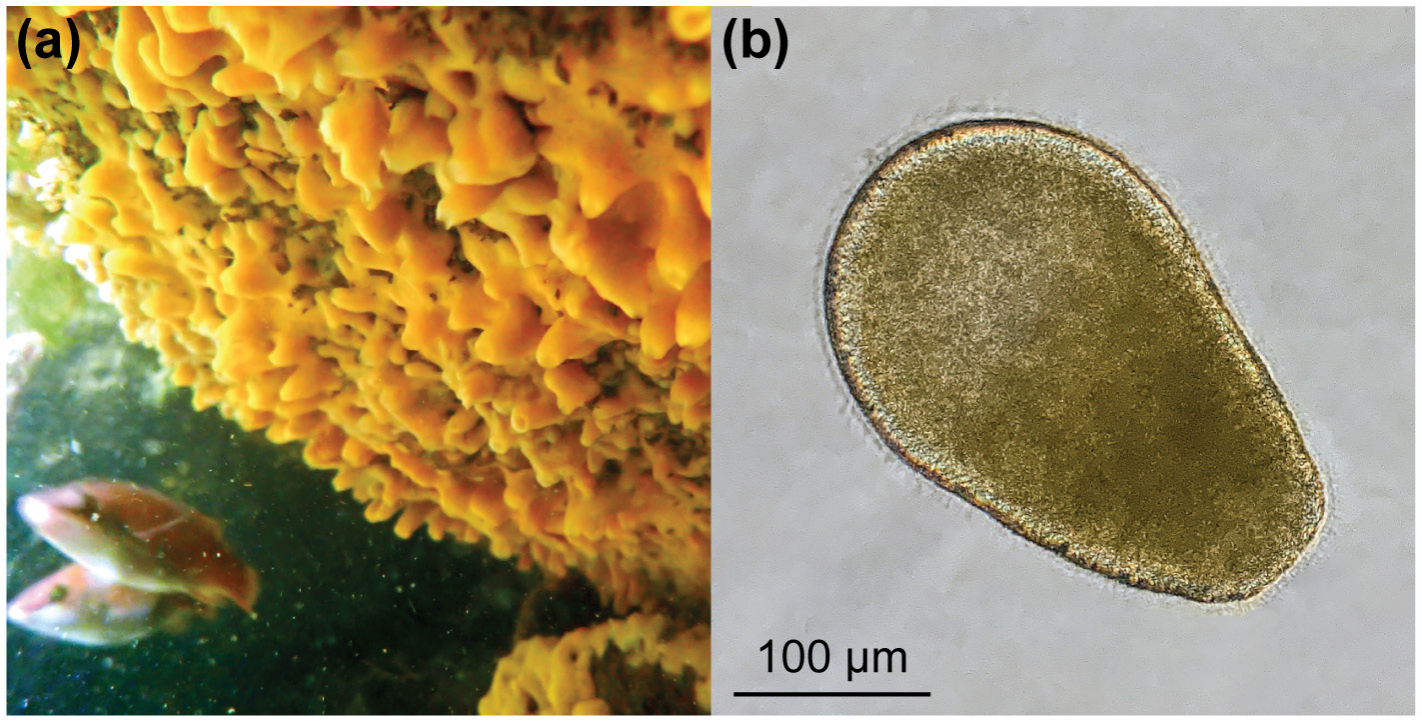
Our study system. (a) The sponge *Halichondria panicea* on the underside of a boulder in the Baltic Sea. (b) A recently released, free‐swimming larva from the viviparous sponge *H*. *panicea*.

One such sponge is *Halichondria panicea* (Figure 2a), a coastal species that inhabits large parts of the North Atlantic and has a bacterial community that is dominated (with a 90:1 cell count) by the obligate symbiont *Candidatus* Halichondribacter symbioticus (Erpenbeck et al., 2004; Knobloch et al., 2019, 2020; Schmittmann et al., 2022). This obligate symbiont is specific to *H*. *panicea*, lives a host‐dependent lifestyle, and has the potential for a nutritional exchange with the host (e.g., essential amino acids and sugars) (Knobloch et al., 2020). We used *H*. *panicea* to test whether viviparous offspring have similar numbers of *Ca.* H. symbioticus and total bacteria (i.e., *Ca.* H. symbioticus plus other bacteria) (Figure 2b). Specifically, we estimated the reproductive output of individual sponges and then quantified offspring size, *Ca.* H. symbioticus abundance, and total bacteria using quantitative PCR. We then performed an antibiotic trail and metamorphosis assay to test whether *Ca.* H. symbioticus and the other bacterial members are obligately associated with *H*. *panicea* larvae.

## 2 MATERIALS AND METHODS

### 2.1 Collection of sponge adults and larvae

Adult *H*. *panicea* were collected at ~2 m depth from the breakwaters at Schilksee Strandbad (Kiel, Germany; 54.424772, 10.175033; Figure 2a) during May and June of 2020 (*n* = 30; from two collections), 2021 (*n* = 29; from two collections), and 2022 (*n* = 14; from one collection) because this is their known reproductive period in the area (Barthel, 1986; Witte et al., 1994). Sponges were gently removed from boulders using a paint scraper, kept fully submerged in seawater, and transferred to flow‐through aquaria at the GEOMAR Helmholtz Center for Ocean Research (Kiel, Germany) within 1 h. Sponges were trimmed to an area of ~45 cm^2^, positioned on terra cotta plates, and mesh traps were placed around each individual (Figure S1A) (Lindquist et al., 1997). All sponges were provided at least 2 days to acclimate to the flow‐through aquaria.

In 2020, tissues were sampled from each adult before transferring them to aquaria as well as after spawning finished. Sponges were inspected for larvae every other day using a dissecting microscope (Motic, series SMZ168), and larvae were then quantified daily for each adult for ~3 weeks once larvae were observed (Figure 2b). Our sampling period overlapped completely with the known period of larval release for this population (Barthel, 1986; Witte et al., 1994). However, we acknowledge that sponges might have started spawning in the field before our collection and, thus, we may have not collected every larva from each individual. Larvae were collected from the bottle (~100 mL) component of each trap as well as from the surface off each parental sponge (~100 mL by pipetting) (Figure S1A). Tissues were preserved in RNAlater at 4°C for 24 h and then at −80°C for long‐term storage.

Consistent with a previous study (Elliott et al., 2004), *H*. *panicea* larvae were found predominately on the adult surface in 2020 and, thus, we used a modified mesh tipi in 2021 and 2022 to restrict the flow around the adults (Figure S1B). We sampled ~100 mL of seawater by pipette from the surface of each parental sponge on each day for up to 11 days (a total of 2 weeks in the aquaria when the acclimation period was included), after which new individuals were collected and monitored. Surface water from each sponge was then transferred to separate jars and the number of larvae was quantified for each adult using a dissecting microscope (Motic, series SMZ168).

### 2.2 Antibiotic exposure experiment

In June 2021, subsets of larvae were treated with antibiotics to test whether Ca. H. symbioticus and total bacteria could be removed and whether metamorphosis is impacted. Larvae were pooled from all reproductive adults on each of those days and were transferred to petri dishes in a gnotobiotic chamber (Schmittmann et al., 2022). Larvae (*n* = 50) were exposed to either control conditions (0.22 μm filtered sea water) or an antibiotic cocktail (Streptomycin at 25 μg/mL, Penicillin at 10 U/mL, and Rifampicin 30 μg/mL). This antibiotic cocktail was derived from, but is not identical to, Schmittmann et al. (2022) because all larvae stopped swimming and experienced developmental abnormalities within 24 h of being exposed to the antibiotic cocktail that was used previously on adult *H*. *panicea* (Schmittmann et al., 2022). Therefore, antibiotically‐treated larvae presented here were all actively swimming and did not have any developmental abnormalities and, thus, this perturbation was sub‐lethal. Technical replicates were sampled at 24 h and 48 h (*n* = 4). Larvae were collected in sterile 1.5 mL Eppendorf tubes and stored at −80°C for long‐term storage, while larvae that metamorphosed (i.e., juveniles) were counted using a dissecting microscope (Motic, series SMZ168).

### 2.3 DNA extraction and quantitative PCR

Total DNA was extracted according to the manufacturer's protocol for the DNeasy® Blood & Tissue Mini Kit (Qiagen) from ~90 mg of adult tissue, pooled larvae (clutches per adult) that were collected over multiple weeks from individual adults, and larvae following experimental treatment. Total DNA was then quantified using the dsDNA BR Assay Kits for the Qubit Fluorometer (Thermo Fisher Scientific) following the manufacturer's protocol. DNA yield increased proportionally with the total number of larvae (Figure S2A; *F*_1,10_ = 41.91, *p* < .0001; *R* ^2^ = 0.807), which supports that this DNA extraction method was sufficient across this fecundity range.

*Ca.* H. symbioticus was quantified using species‐specific primers for the 16S rRNA gene (F: CGCGGATGGTAGAGATACCG; R: TGTCCCCAACTGAATGCTGG; 148 bp; Schmittmann et al., 2022), the total bacterial community was quantified using eubacterial primers for the 16S rRNA gene (F: TGCATGGYTGTCGTCAGCTCG; R: CGTCRTCCCCRCCTTCC; 141 bp; Bahram et al., 2019), and host DNA was quantified using primers for the 18S rRNA gene of Eukaryotes (F: CAGGGTTCGATTCCGTAGAG; R: CCTCCAGTGGATCCTCGTTA; 185 bp; Bayer et al., 2014). A standard curve was prepared by running 50 μL PCR reactions, as would be prepared for quantitative PCR (qPCR). Gel electrophoresis was performed on these PCR products, which were then cut from the gel using a sterile scalpel and cleaned by following the manufacturers' protocol in the NucleoSpin Gel and PCR Clean‐up kit (Marcherey Nagel). A 10‐fold dilution series from 10^−9^ to 10^−3^ ng/μL of DNA was then prepared and the copy number was determined based on the DNA concentration and fragment length.

Each qPCR reaction (totaling 20 μL) was performed in triplicate using 4 μL of template (at 2.5 ng/μL for adult‐clutch comparisons and 0.375 ng/μL for all others; Figure S2B,C), 10 μL of the Maxima SYBR Green qPCR Master Mix (Thermofisher), 0.1 μL of forward and reverse primers (total of 250 nM), and 5.8 μL of DNAase and RNAase‐free water. The following cycle parameters were used on a CFX Connect Real‐Time PCR Detection System (Bio‐Rad) thermocycler: 96°C for 2 min, followed by 40 cycles of 94°C for 30 s, 60°C for 30 s, and 72°C for 60 s, and a melting curve analysis was conducted by increasing temperature from 60°C to 95°C during 5 s. Absolute copy number was then calculated according to the standard curve.

### 2.4 Statistics

Quantitative estimates of reproductive output for each adult sponge were normalized by log transformation. Log‐transformed data were compared between years using a one‐way analysis of variance (ANOVA), a linear regression was used for spawning duration and total sponges, and a second‐order polynomial (quadratic) for day number. A linear regression was also used to compare day number and total sponges. Quantitative estimates of *Ca.* H. symbioticus, the total bacterial community, and host cells (via the 18S rRNA gene) were standardized to 1 ng of DNA and then normalized by log transformation. Log‐transformed data were compared between life stages (adults and larvae) using a paired *t*‐test for each data type.

We used a series of linear regressions to test whether *Ca.* H. symbioticus and the total bacterial community were consistent between offspring from different individual sponges. First, we compared the log‐transformed quantifications for total larvae and *Ca.* H. symbioticus or the total bacterial community abundance per larva (*n* = 10). Second, we compared the log‐transformed quantifications to host cells per larva to total larvae (*n* = 5). Third, we compared the log‐transformed quantifications of host cells to *Ca.* H. symbioticus or the total bacterial community abundance per larva (*n* = 5). The reduction in biological replications in the latter linear regressions was due to an insufficient quantity of DNA. We then used paired *t*‐tests to determine whether log‐transformed quantifications of *Ca*. H. symbioticus or the total bacterial community differed for adults before and after spawning. Separate one‐way ANOVAs (with Tukey's pairwise comparisons) were used to test whether *Ca.* H. symbioticus or the total bacterial community were reduced in larvae following an antibiotic treatment. A paired *t*‐test was used to determine whether settlement at 48 h was affected by this experimental exposure.

All analyses were performed in Prism (v. 9.0.0). Graphs were created in Prism and then stylized using Adobe Illustrator.

## 3 RESULTS

### 3.1 Reproductive output and symbiont quantification

**FIGURE 3:**
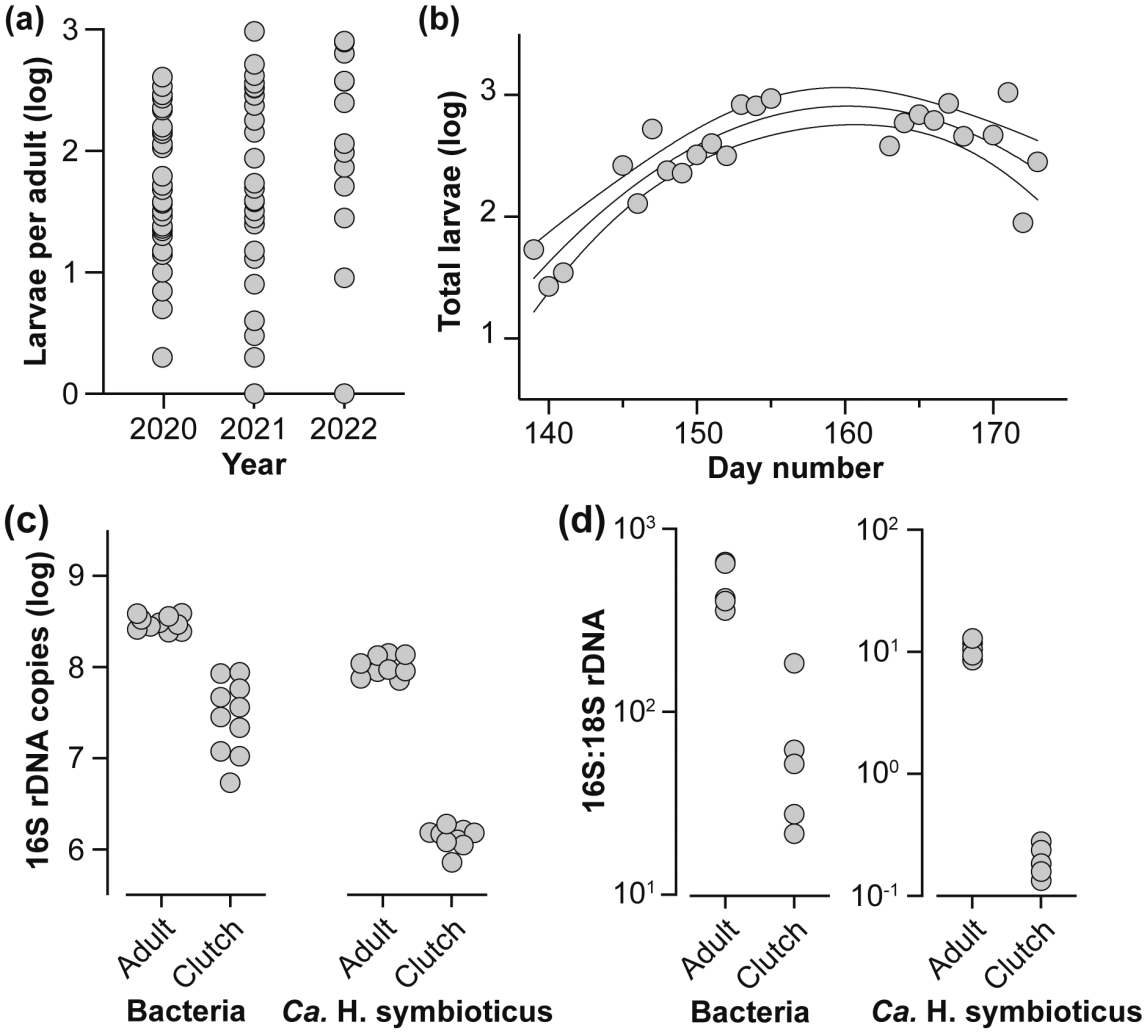
Reproductive output and symbiont transmission by *Halichondria panicea*. (a) Clutch size of adult *H*. *panicea* was consistent for three consecutive years. (b) The reproductive output of *H*. *panicea* was dynamic over the spawning season, such that there was a gradual increase in total larvae from mid‐May (day number 140) until early June (day number 160) and a decline thereafter (average ± 95% confidence intervals). (c) Estimated number of 16S rDNA copies for the total bacterial community (left) and *Candidatus* Halichondribacter symbioticus (right) in adults and their clutch. In both cases, the total 16S rDNA copies were significantly less in the clutch than in the adult. (d) Estimated ratio of 16S to 18S rDNA copies for the total bacterial community (left) and *Ca.* H. symbioticus (right) in adults and their clutch. In both cases, the offspring had fewer total bacteria and *Ca.* H. symbioticus per host cell than their respective adult. All data were log‐transformed for normalization.

The reproductive output by this population of *H*. *panicea* was consistent across years (one‐way ANOVA, *F* = 1.122, *p* = .332; Figure 3a; Table S1). The number of larvae released by individual adults spanned three orders of magnitude, and those that released larvae over a longer duration tended to have a greater reproductive output (*F*_1,40_ = 50.46, *p* < .0001; *R* ^2^ = .558; Figure 3a; Figure S3A,B). Consistent with previous reports (Barthel, 1986; Witte et al., 1994), the reproductive output by this population increased gradually with sea surface temperature from mid‐May (day number ~ 140) until early June (day number ~ 160), after which there was a steady decline in reproductive output despite a continued increase in sea surface temperature (SS: 1.129, *R* ^2^ = 0.753, df = 21; Figure 3b; Figure S3C). The relationship between day number and reproductive output was unlikely to be a byproduct of sampling because our sampling effort increased linearly with day number (*F*_1,23_ = 6.543, *p* = .018; *R* ^2^ = 0.222; Figure S3D) and reproductive output increased linearly with the total adults that were sampled (*F*_1,23_ = 16.21, *p* = .0005; *R* ^2^ = 0.413; Figure S3E).

The estimated number of 16S rDNA copies per ng of DNA for *Ca.* H. symbioticus was significantly (81.3x) higher in adults than in their clutch (paired *t*‐test, *p* < .0001; Figure 3c; Table S2). This pattern was also observed for the total bacterial community, whereby adults had 7.9x more total bacteria than the clutch (paired *t*‐test, *p* < .0001; Figure 3c). Total 16S rDNA copies for *Ca.* H. symbioticus and the total bacterial community outnumbered the 18S rDNA copies of *H*. *panicea* for both adults and the clutch. Adults had 65.5x more *Ca.* H. symbioticus (paired *t*‐test, *p* = .0002) and 7.2x more bacteria (paired *t*‐test, *p* < .0001) per 18S rDNA copy than their clutch (Figure 3d; Table S3).

### 3.2 Provisioning of *Ca.* H. symbioticus

**FIGURE 4:**
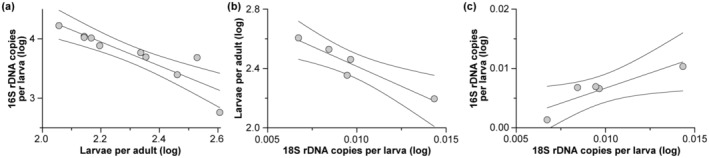
Maternal provisioning of *Candidatus* Halichondribacter symbioticus. (a) The reproductive output of *Halichondria panicea* was estimated by the number of larvae per adult and the number of *Ca.* H. symbioticus was then quantified from each clutch. A negative correlation between reproductive output and *Ca.* H. symbioticus abundance per larva (average ± 95% confidence intervals), such that larvae from more fecund adults had less *Ca.* H. symbioticus on average than larvae from less fecund adults. (b) A negative correlation between reproductive output and larval size (i.e., 18S rDNA copies per larva), such that more fecund adult *H*. *panicea* tended to produce smaller larvae than less fecund adult larva (average ± 95% confidence intervals). (c) A positive correlation between larval size and *Ca.* H. symbioticus abundance, whereby smaller larvae had less *Ca.* H. symbioticus per larva than that of larger larvae (average ± 95% confidence intervals). All data types were log‐transformed for normalization.

We used the quantitative estimates of reproductive output for *H*. *panicea* from 2020, host cells (a proxy for offspring size), *Ca.* H. symbioticus, and total bacteria to test whether offspring between clutches have quantitatively similar numbers of symbionts (Figure 3). We did not observe that offspring between different *H*. *panicea* individuals had similar numbers of *Ca.* H. symbioticus. Instead, we observed a negative correlation between fecundity and *Ca.* H. symbioticus abundance per larva (*F*_1,8_ = 32.20, *p* = .0005; *R* ^2^ = 0.801), such that larvae from more fecund adults had less *Ca.* H. symbioticus on average than larvae from less fecund adults (Figure 4a). This pattern, however, was not observed for the total bacterial community (*F*_1,8_ = 3.53, *p* = .097; *R* ^2^ = 0.306; Figure S4A). Moreover, we observed a negative correlation between reproductive output and larval size as well as a positive correlation between larval size and *Ca.* H. symbioticus abundance, such that more fecund sponges had smaller larvae with less *Ca.* H. symbioticus per larva than that of less fecund sponges (larval size vs. reproductive output: *F*_1,3_ = 27.21, *p* = .014, *R* ^2^ = 0.901; larval size vs. *Ca.* H. symbioticus: *F*_1,3_ = 12.17, *p* = .039, *R* ^2^ = 0.802; Figure 4b,c). This pattern, again, was not observed for the total bacterial community (larval size vs. total bacteria: *F*_1,7_ = 4.253, *p* = .078, *R* ^2^ = 0.378; Figure S4B).

**FIGURE 5:**
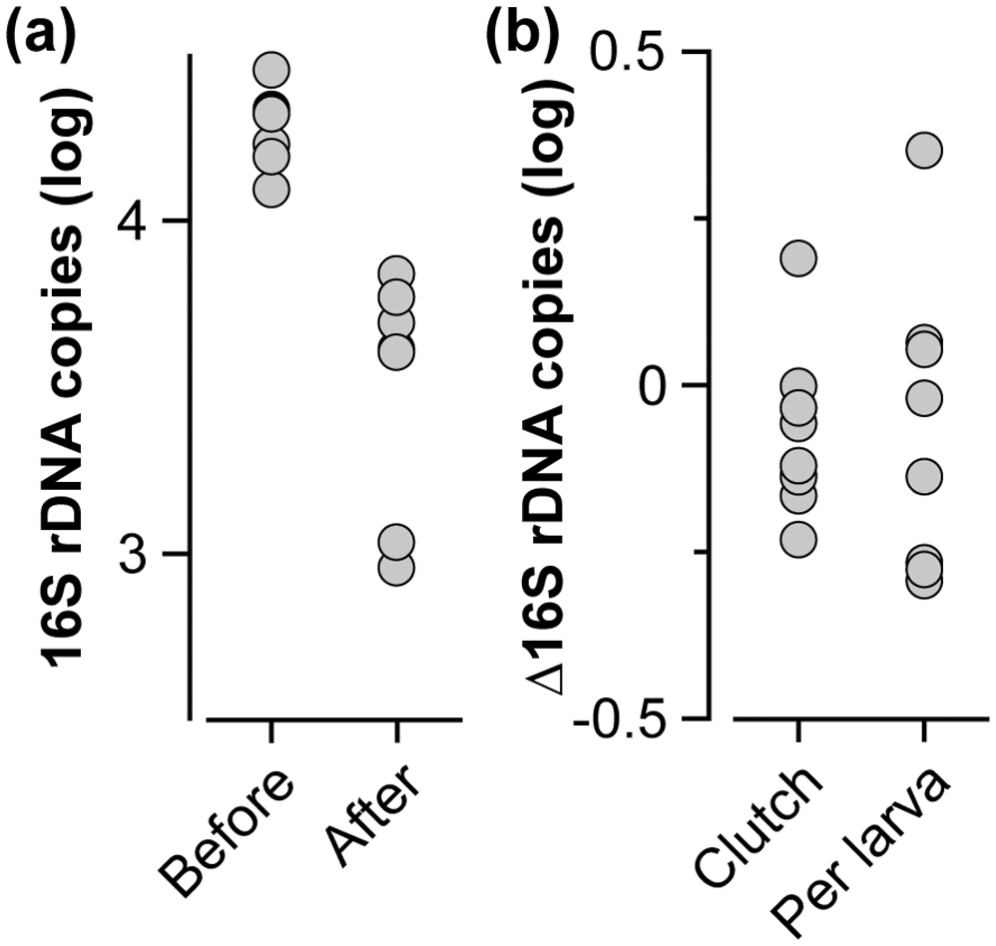
A maternal pool of *Candidatus* Halichondribacter symbioticus. (a) The abundance of *Ca.* H. symbioticus was relatively consistent within adult *H*. *panicea* before spawning and significantly decreased after spawning. (b) Variation in the maternal pool of *Ca.* H. symbioticus was 26.6%, while that per larva was 57.8% higher. All data types were log‐transformed for normalization.

The investment of resources into reproduction is finite (Smith & Fretwell, 1974). If *Ca.* H. symbioticus is provisioned as part of this maternal investment, then it may be hypothesized that there is a maternal pool of *Ca.* H. symbioticus for reproduction, that this maternal pool is depleted following reproduction, and that there is less variation in this maternal pool than the provisioned offspring. Adults had less *Ca.* H. symbioticus after spawning than they did before spawning, and this pattern was also observed for the total bacterial community (paired *t*‐test, *p* < .0001 for both; Figure 5a; Figure S4C; Table S4). Moreover, variation in the maternal pool of *Ca.* H. symbioticus and the total bacterial community was, on average, 26.6% and 40.6% of a magnitude, respectively (Figure 5b; Figure S4D). Variation per larva was 57.8% higher for *Ca.* H. symbioticus and 17.8% higher for the total bacterial community than in the adults (Figure 5b; Figure S4D).

### 3.3 Stability of *Ca.* H. symbioticus

**FIGURE 6:**
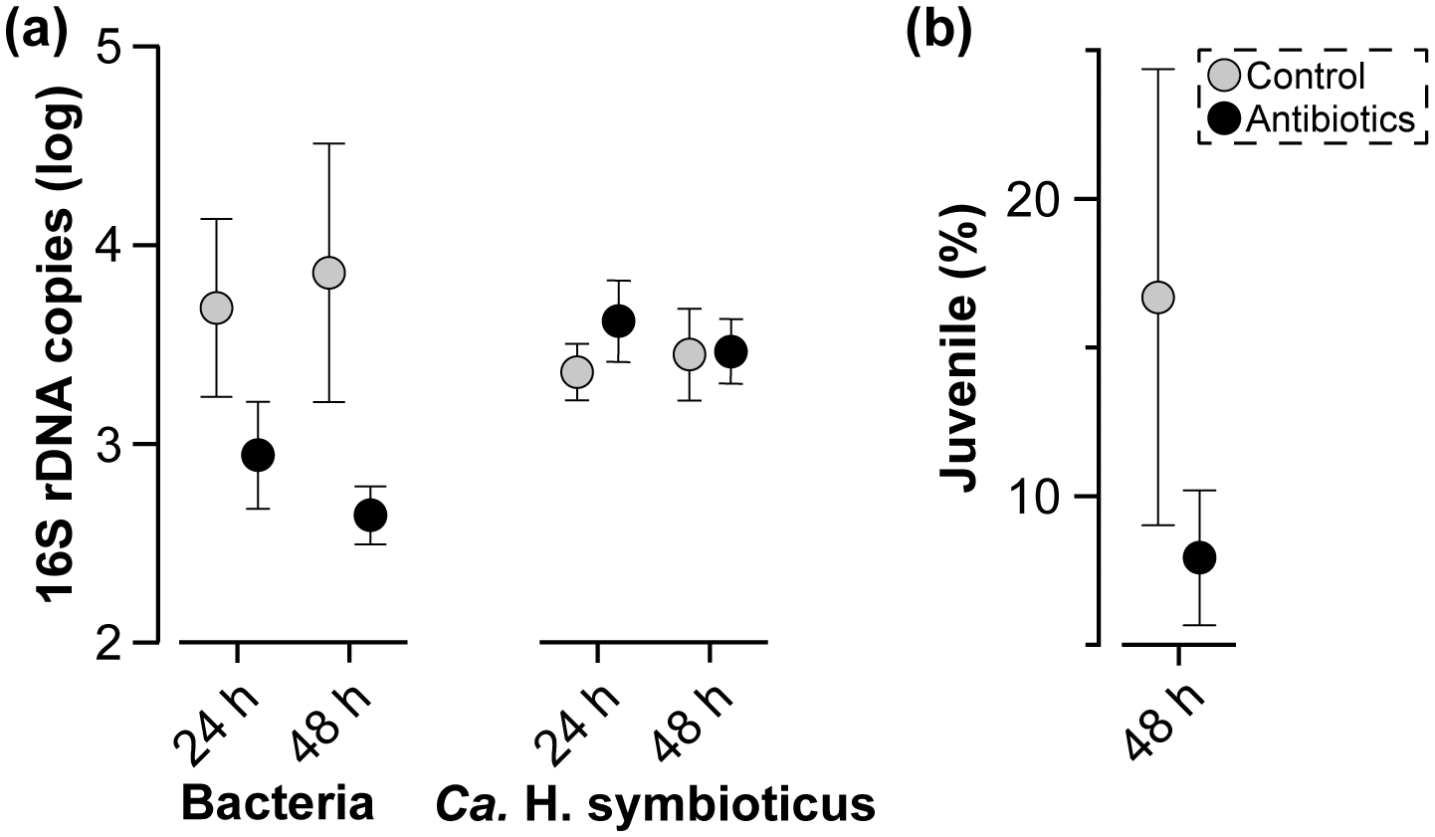
Stability of *Candidatus* Halichondribacter symbioticus in larvae. (a) The total bacterial community was reduced in *Halichondria panicea* larvae following 48 h of antibiotics, while the abundance of *Ca.* H. symbioticus could not be reduced (average ± standard deviation). These data were log‐transformed for normalization. (b) The reduction in the total bacterial community did not significantly affect the ability of *H*. *panicea* larvae to settle and undergo metamorphosis (average ± standard deviation).

If the provisioning of *Ca.* H. symbioticus is part of the reproductive life history for *H*. *panicea*, then it may be expected that the abundance of this symbiont is not easily perturbed and that any reduction in abundance would impact offspring performance. Treating *H*. *panicea* larvae with an antibiotic cocktail experimentally reduced the abundance of the total bacterial community, but did not reduce the abundance of *Ca.* H. symbioticus (one‐way ANOVA for total bacteria, *F* = 5.665, *p* = .012; Tukey's, control vs. antibiotics at 48 h: *p* = .01, all other comparisons: *p* > 0.05; one‐way ANOVA for *Ca.* H. symbioticus, *F* = 1.346, *p* = .306; Figure 6a; Table S5). A reduction in the total bacterial community did not significantly affect the ability of *H*. *panicea* larvae to undergo metamorphosis (paired *t*‐test, *p* = .137; Figure 6b; Table S5).

## 4 DISCUSSION

Quantitative strategies to transmit maternal microbes to offspring could be stochastic, consistent, or provisioned (Figure 1). Examples to date stem from oviparous species and suggest that offspring are provided a consistent number of maternal microbes (Flórez & Kaltenpoth, 2017; Mira & Moran, 2002; Pons et al., 2022). By quantifying the number and size of *H*. *panicea* larvae as well as the number of *Ca.* H. symbioticus, we do not support the premise that offspring of this viviparous sponge are provided a similar number of maternal symbionts. *H*. *panicea* appears to have a maternal pool of bacteria, and *Ca.* H. symbioticus is partitioned proportionally to the reproductive output and allometrically by offspring size, while other members of the bacterial community were more stochastic. A trade‐off between offspring size and number is, by definition, maternal provisioning and parallel differences in *Ca.* H. symbioticus abundance would suggest that this symbiont is also provisioned (Carrier & McAlister, 2022; Kieft & Simmons, 2015; Smith & Fretwell, 1974).

Mothers from diverse metazoan lineages are known to influence the development of their offspring through gene transfer and maternal effects (Marshall & Uller, 2007; Mousseau & Fox, 1998; Wolfe & Wade, 2009). These effects are broadly classified and most notably include the resources that are provisioned to an offspring during internal gestation or to the egg of external developers. Typically, these resources include carbohydrates, lipids, mRNAs, and proteins, and the quantity and composition of these resources can have a profound impact on offspring survival, performance, and, thus, fitness (Byrne et al., 2008; Einum & Fleming, 2000; Jaeckle, 1995; Moran & McAlister, 2009). Total maternal investment in reproduction, however, is finite and selection balances offspring size and number (Fox et al., 1997; Smith & Fretwell, 1974). As such, energetic content scales with offspring size and this increases the likelihood that larger offspring survive (Jaeckle, 1995; McEdward & Morgan, 2001; Moran et al., 2013). The trade‐off between *H*. *panicea* reproduction and *Ca.* H. symbioticus provides support for maternal provisioning in sponges and for an obligate symbiont to fall within the framework of maternal provisioning.

The total bacterial community provided to *H*. *panicea* larvae was inconsistent with this premise. Similar to animal hosts from diverse lineages, the bacterial community associated with sponges is a mix of generalists that are partially formed by neutral processes and a fraction of these microbes are thought to be stochastically transmitted to the offspring (Björk et al., 2019; Sieber et al., 2019; Thomas et al., 2016). It has been postulated that some maternally transmitted microbes provide little to no fitness to the sponge (Björk et al., 2019; Rodrigues de Oliveira et al., 2020). Microbes with little to no benefit to host fitness are unlikely to co‐vary with patterns and strategies of host reproduction (Frank, 1996; Wade, 2014). Supporting this, the total bacterial community did not co‐vary with *H*. *panicea* reproduction, and these bacteria could be depleted upon antibiotic treatment without impacting the likelihood of metamorphosis. Additional fitness measures for juveniles (e.g., metabolism and physiology) were not tested due to long‐standing challenges in maintaining this life stage for sponges under laboratory conditions. A minimal contribution towards host fitness by bacteria other than *Ca.* H. symbioticus may explain why the total bacterial community was not provisioned as part of the reproductive life history of *H*. *panicea*.

The inability to perturb *Ca.* H. symbioticus abundance in larvae, as well as adults (Schmittmann et al., 2022), and the pattern of maternal provisioning provides preliminary and indirect evidence that this symbiont could benefit maternal and offspring fitness. Therefore, this needs to be tested explicitly by disrupting the transmission of *Ca.* H. symbioticus and subsequently quantifying developmental rate, survivorship, and reproductive output (Marshall & Uller, 2007; Mousseau & Fox, 1998; Wolfe & Wade, 2009). The inability to perturb *Ca.* H. symbioticus abundance could also reflect that this obligate symbiont is compartmentalized and more isolated from external influence. The cellular structure remains unknown for *Ca.* H. symbioticus, but genomic and experimental evidence suggests that the abundance of this obligate symbiont is under host control (Knobloch et al., 2020; Schmittmann et al., 2022). If *Ca.* H. symbioticus abundance is under host control and correlates with the reproductive life history of *H*. *panicea*, then the maternal provisioning of this obligate symbiont may be an active process.

If *Ca.* H. symbioticus is an active and advantageous component of maternal provisioning, then offspring with more *Ca.* H. symbioticus could be fitter than those with less *Ca.* H. symbioticus. Priming offspring with, and potentially by, *Ca.* H. symbioticus could be part of a bet‐hedging strategy to maximize maternal fitness in an unpredictable environment, such as that of the Baltic Sea (Bruijning et al., 2022; Carrier & McAlister, 2022; Kaiwa et al., 2014; Marshall et al., 2008; Marshall & Keough, 2003; Marshall & Uller, 2007). The determinate(s) of maternal provisioning in *H*. *panicea* remain unknown. Our collections included adult sponges of all sizes—a coarse correlate for age (McMurray et al., 2008)—from one location and, thus, we hypothesize that adult age is a factor in maternal provisioning by *H*. *panicea* and of *Ca.* H. symbioticus. Comparison of developmentally synchronized adults would enable host and microbial factors of maternal provisioning to be identified, but this is not yet feasible in *H*. *panicea* or sponges in general.

Evolutionary ecology is centered around the examination of individual life‐history characters, which are increasingly recognized to be dependent on microbial symbionts (Bates et al., 2006; Carrier & Reitzel, 2018; Lynch & Hsiao, 2019; McFall‐Ngai et al., 2013; McFall‐Ngai & Ruby, 1991). Provided that host‐microbe symbioses are units of biological organization upon which multilevel selection acts (Bordenstein & Theis, 2015; Zilber‐Rosenberg & Rosenberg, 2008), the ways that mothers impact offspring survival, performance, and fitness is anticipated to include the provisioning of maternal microbes to offspring (Bright & Bulgheresi, 2010; Bruijning et al., 2022; Carrier & Bosch, 2022; Carrier & McAlister, 2022; McFall‐Ngai, 2002; Metcalf et al., 2019; Nyholm, 2020; Pons et al., 2022). Our quantitative estimates of *H*. *panicea* reproduction and *Ca.* H. symbioticus abundance provides an example of where an obligate symbiont falls within the framework of maternal provisioning. This raises questions on how widespread this phenomenon is in host–microbe symbioses, whether the provisioned microbes benefit maternal and offspring fitness, if maternal provisioning of microbes is an active or passive process, and which host, microbial, and environmental factors influence the maternal provisioning of obligate symbionts.

## AUTHOR CONTRIBUTIONS

**Tyler J Carrier:** Conceptualization (equal); data curation (lead); formal analysis (lead); investigation (equal); methodology (equal); writing – original draft (lead); writing – review and editing (lead). **Lara Schmittmann:** Conceptualization (equal); investigation (lead); methodology (equal); writing – review and editing (equal). **Sabrina Jung:** Investigation (equal); methodology (equal); writing – review and editing (supporting). **Lucía Pita:** Conceptualization (equal); investigation (equal); methodology (equal); writing – review and editing (equal). **Ute Hentschel:** Conceptualization (equal); funding acquisition (lead); project administration (lead); resources (lead); supervision (lead); writing – review and editing (equal).

## CONFLICT OF INTEREST STATEMENT

We declare that we have no competing interests.

## WOA INSTITUTION

HELMHOLTZ‐ZENTRUM FUR OZEANFORSCHUNG KIELConsortia Name: Projekt DEAL.

## Supporting information


Appendix S1

## Data Availability

Quantitative estimates for reproductive effort (fecundity), total host cells (18S rDNA), and symbiont cells (16S rDNA) are provided in the Appendix S1.
