# Supplementary material for: Maternal provisioning of an obligate symbiont in a sponge
Source: Ecol Evol. 2023 May 2;13(5):e10012. doi: 10.1002/ece3.10012 (PMC10154371; doi:10.1002/ece3.10012)
Supplement: Supplementary file 1 — Appendix S1 [file ECE3-13-e10012-s001.pdf]

Appendix for

Maternal provisioning of an obligate symbiont in a sponge

Tyler J. Carrier[1,2,*], Lara Schmittmann[1], Sabrina Jung[1], Lucía Pita[1,3], and Ute Hentschel[1,2,*]

[1] GEOMAR Helmholtz Center for Ocean Research, Kiel, Germany

[2] Zoological Institute, Christian-Albrechts University of Kiel, Kiel, Germany

[3] Department Marine Biology and Oceanography, Institute of Marine Sciences (ICM-CSIC), Barcelona, Spain

[*] Corresponding author: tcarrier@geomar.de

This PDF file includes:

Figure S1 to S4

Table S1 to S5

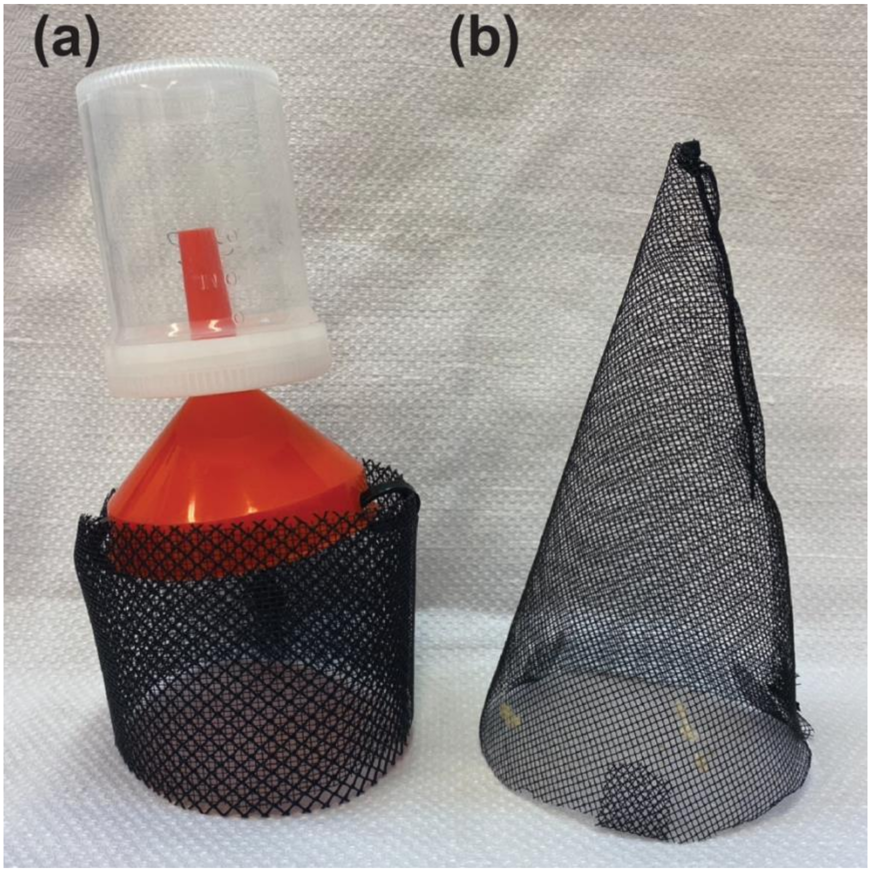

Figure S1: Contraptions used to contain *Halichondria panicea* larvae. (A) Larvae were collected from the bottle component of this trap as well as from the surface off each parental sponge in 2020. (B) Larvae were found predominately on the adult surface and, thus, we used this modified mesh tipi to restrict the flow around the adults and sample seawater from the surface of each adult in 2021 and 2022.

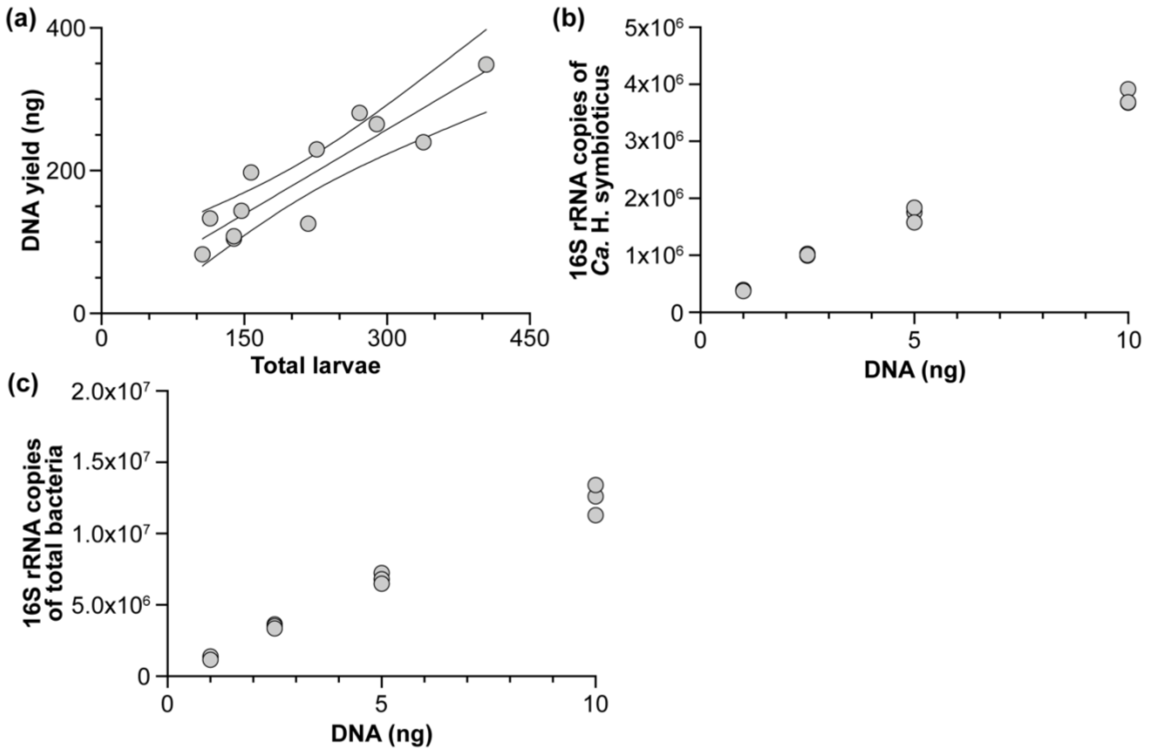

Figure S2: Molecular parameters of *Halichondria panicea* larvae and *Candidatus* Halichondribacter symbioticus. (A) The DNA yield increased proportionally with the total number of larvae (average ± 95% confidence intervals), such that each larva had on average 0.89 ng of DNA. (B) The detection of *Ca.* H. symbioticus increased proportionally with DNA input and justified using 1.5 ng of total DNA per quantitative PCR reaction were sufficient for reliably detecting and used for all reactions. (C) The detection of total bacteria increased proportionally with DNA input and justified using 1.5 ng of total DNA per quantitative PCR reaction were sufficient for reliably detecting and used for all reactions.

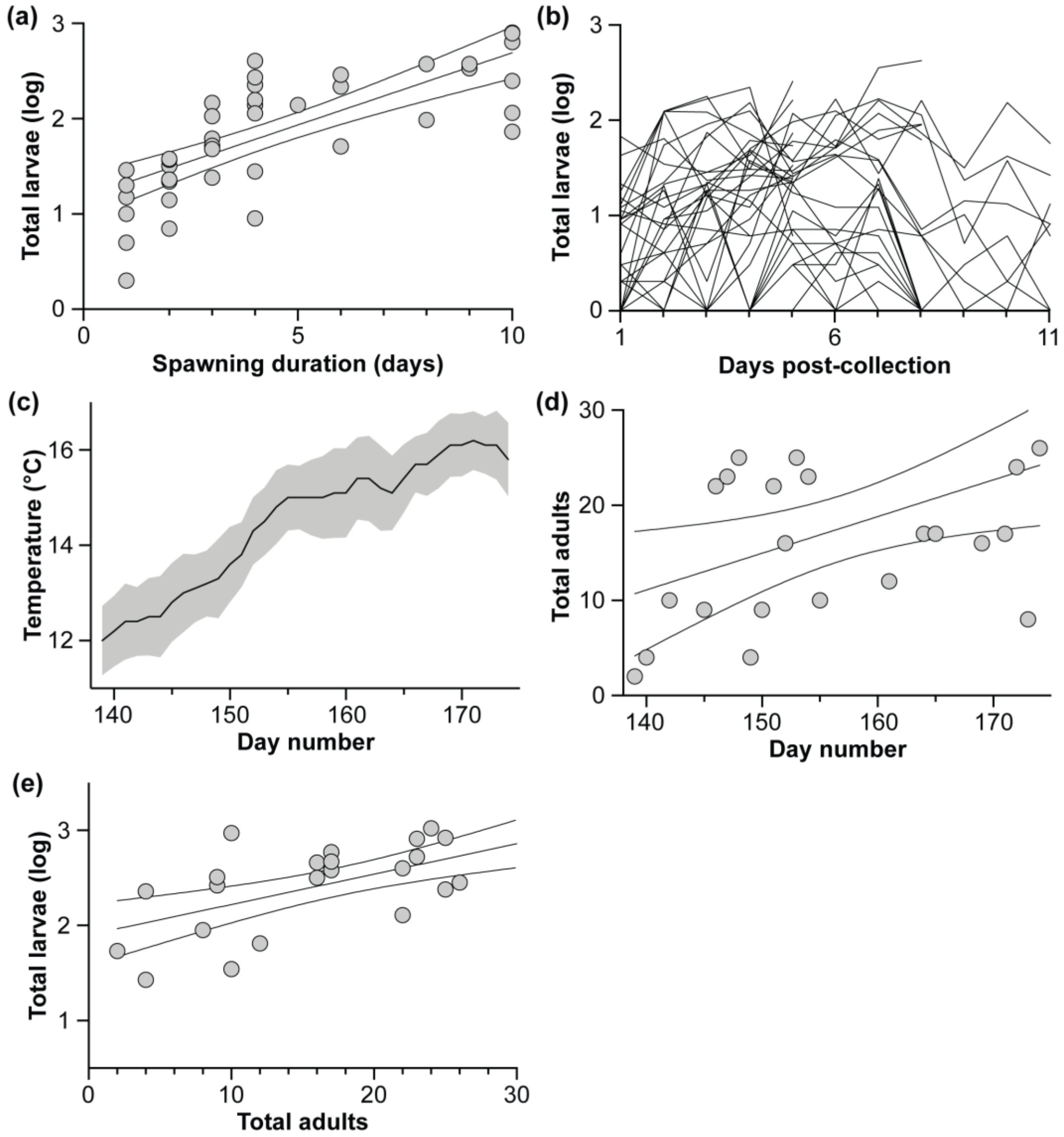

Figure S3: Reproductive output of *Halichondria panicea*. (A) Estimation of the clutch size for individual *H. panicea* and how this output relates to the duration that an individual sponge releases its larvae (average ± 95% confidence intervals). (B) The estimated number of released larvae for 54 individuals (*i.e.*, all from 2021 and 2022) for up to 11 days after collection. (C) Daily mean temperatures between 19 May and 23 June for the years 1997–2021 (black line: daily mean averages, grey area: 95% confidence intervals) recorded at 1.5 m depth from the Kiel Fjord (54°19'46.0"N, 10°08'58.3"E). These data for 2022 are not yet available from the GEOMAR Helmholtz Centre for Ocean Research and, thus, are not included here. (D) The positive relationship between day-number of the year and total sponges that were being sampled for larvae (average ± 95% confidence intervals). (E) The positive relationship between total adults sampled and total larvae (average ± 95% confidence intervals). Most data types were log transformed for normalization.

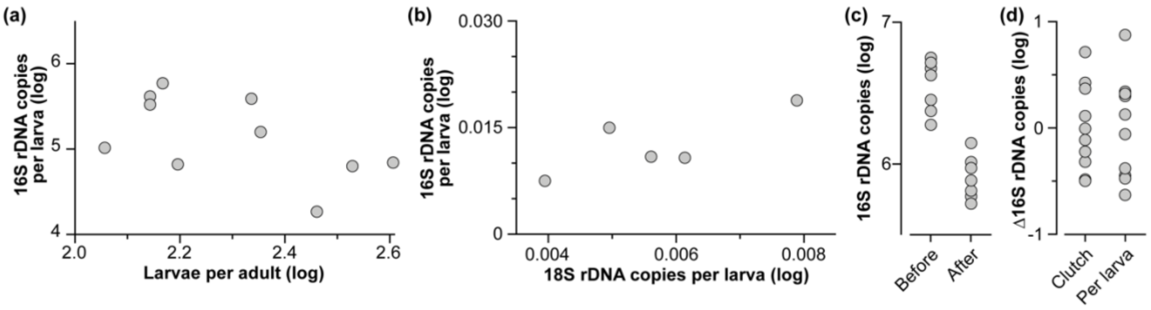

Figure S4: No provisioning of the total bacterial community. (A) The number of larvae was estimated for adult *Halichondria panicea* and the number of total bacteria was then quantified from each clutch. We observed a negative, but non-significant, trend between fecundity and total bacteria per larva. (B) We observed a negative, but non-significant, tend between host cells and total bacterial. (C) Total bacteria were quantified for adult *H. panicea* before and after spawning, and we observed that adults have less bacteria after spawning than they did before spawning. (D) Variation in total bacteria between clutches and based on per larva averages. All data types were log transformed for normalization.

Table S1. Counts of sponge larvae over three spawning seasons.

2020			2021			2022		
**Date**	**Sponge**	**Larvae**	**Date**	**Sponge**	**Larvae**	**Date**	**Sponge**	**Larvae**
19.05.2020	1	4	26.05.2021	1	15	10.06.2022	1	--
19.05.2020	15	50	26.05.2021	2	0	10.06.2022	2	12
20.05.2020	1	6	26.05.2021	3	0	10.06.2022	3	4
20.05.2020	3	12	26.05.2021	4	0	10.06.2022	4	9
20.05.2020	7	5	26.05.2021	5	9	10.06.2022	5	2
20.05.2020	19	4	26.05.2021	6	0	10.06.2022	6	2
22.05.2020	1	8	26.05.2021	7	0	10.06.2022	7	0
22.05.2020	2	2	26.05.2021	8	1	10.06.2022	8	0
22.05.2020	6	3	26.05.2021	9	0	10.06.2022	9	0
22.05.2020	7	7	26.05.2021	10	0	10.06.2022	10	--
22.05.2020	8	1	26.05.2021	11	0	10.06.2022	11	0
22.05.2020	10	6	26.05.2021	12	0	10.06.2022	12	1
22.05.2020	11	2	26.05.2021	13	0	10.06.2022	13	--
22.05.2020	15	2	26.05.2021	14	0	10.06.2022	14	21
22.05.2020	17	3	26.05.2021	15	0	10.06.2022	15	13
22.05.2020	18	1	26.05.2021	16	0	10.06.2022	16	--
25.05.2020	1	30	27.05.2021	1	7	10.06.2022	17	--
25.05.2020	1	20	27.05.2021	2	0	13.06.2022	1	21
25.05.2020	3	40	27.05.2021	3	0	13.06.2022	2	22
25.05.2020	7	40	27.05.2021	4	0	13.06.2022	3	54
25.05.2020	9	2	27.05.2021	5	20	13.06.2022	4	15
25.05.2020	11	12	27.05.2021	6	0	13.06.2022	5	0
25.05.2020	15	50	27.05.2021	7	0	13.06.2022	6	1

25.05.2020	17	20	27.05.2021	8	9	13.06.2022	7	0
25.05.2020	19	50	27.05.2021	9	0	13.06.2022	8	9
26.05.2020	2	20	27.05.2021	10	0	13.06.2022	9	2
26.05.2020	4	11	27.05.2021	11	2	13.06.2022	10	0
26.05.2020	6	10	27.05.2021	12	0	13.06.2022	11	0
26.05.2020	8	6	27.05.2021	13	0	13.06.2022	12	0
26.05.2020	10	40	27.05.2021	14	0	13.06.2022	13	2
26.05.2020	12	16	27.05.2021	15	0	13.06.2022	14	12
27.05.2020	1	90	27.05.2021	16	0	13.06.2022	15	121
27.05.2020	15	29	28.05.2021	1	18	13.06.2022	16	122
27.05.2020	19	44	28.05.2021	2	0	13.06.2022	17	0
27.05.2020	23	109	28.05.2021	3	0	14.06.2022	1	28
27.05.2020	27	64	28.05.2021	4	0	14.06.2022	2	66
27.05.2020	31	43	28.05.2021	5	1	14.06.2022	3	89
27.05.2020	37	106	28.05.2021	6	1	14.06.2022	4	21
28.05.2020	10	55	28.05.2021	7	0	14.06.2022	5	15
28.05.2020	16	5	28.05.2021	8	11	14.06.2022	6	22
28.05.2020	22	27	28.05.2021	9	0	14.06.2022	7	0
28.05.2020	24	20	28.05.2021	10	0	14.06.2022	8	16
28.05.2020	26	17	28.05.2021	11	0	14.06.2022	9	0
28.05.2020	28	23	28.05.2021	12	0	14.06.2022	10	0
28.05.2020	32	15	28.05.2021	13	0	14.06.2022	11	1
28.05.2020	34	18	28.05.2021	14	0	14.06.2022	12	0
28.05.2020	38	27	28.05.2021	15	0	14.06.2022	13	18
29.05.2020	1	50	28.05.2021	16	0	14.06.2022	14	21
29.05.2020	23	50	31.05.2021	1	18	14.06.2022	15	119
29.05.2020	27	30	31.05.2021	2	1	14.06.2022	16	174

29.05.2020	37	100	31.05.2021	3	0	14.06.2022	17	0
30.05.2020	10	50	31.05.2021	4	0	15.06.2022	1	30
30.05.2020	12	55	31.05.2021	5	5	15.06.2022	2	150
30.05.2020	19	29	31.05.2021	6	49	15.06.2022	3	123
30.05.2020	22	60	31.05.2021	7	0	15.06.2022	4	48
30.05.2020	24	80	31.05.2021	8	25	15.06.2022	5	46
30.05.2020	26	20	31.05.2021	9	0	15.06.2022	6	14
30.05.2020	28	15	31.05.2021	10	0	15.06.2022	7	0
30.05.2020	34	8	31.05.2021	11	1	15.06.2022	8	6
30.05.2020	38	10	31.05.2021	12	0	15.06.2022	9	1
31.05.2020	1	110	31.05.2021	13	0	15.06.2022	10	0
31.05.2020	23	46	31.05.2021	14	0	15.06.2022	11	0
31.05.2020	27	45	31.05.2021	15	0	15.06.2022	12	0
31.05.2020	31	30	31.05.2021	16	0	15.06.2022	13	0
31.05.2020	35	20	01.06.2021	1	22	15.06.2022	14	1
31.05.2020	37	45	01.06.2021	2	0	15.06.2022	15	70
02.06.2020	6	11	01.06.2021	3	0	15.06.2022	16	35
02.06.2020	10	64	01.06.2021	4	0	15.06.2022	17	0
02.06.2020	12	76	01.06.2021	5	116	16.06.2022	1	29
02.06.2020	19	62	01.06.2021	6	94	16.06.2022	2	27
02.06.2020	22	47	01.06.2021	7	0	16.06.2022	3	36
02.06.2020	24	189	01.06.2021	8	59	16.06.2022	4	25
02.06.2020	34	58	01.06.2021	9	0	16.06.2022	5	17
02.06.2020	36	29	01.06.2021	10	0	16.06.2022	6	5
02.06.2020	38	11	01.06.2021	11	7	16.06.2022	7	0
03.06.2020	1	20	01.06.2021	12	0	16.06.2022	8	7
03.06.2020	4	21	01.06.2021	13	3	16.06.2022	9	5

03.06.2020	21	10	01.06.2021	14	11	16.06.2022	10	0
03.06.2020	23	21	01.06.2021	15	3	16.06.2022	11	0
03.06.2020	27	18	01.06.2021	16	0	16.06.2022	12	0
03.06.2020	31	33	02.06.2021	1	40	16.06.2022	13	3
03.06.2020	37	20	02.06.2021	2	1	16.06.2022	14	21
04.06.2020	10	2	02.06.2021	3	0	16.06.2022	15	37
04.06.2020	18	36	02.06.2021	4	0	16.06.2022	16	21
04.06.2020	19	100	02.06.2021	5	52	16.06.2022	17	0
04.06.2020	22	5	02.06.2021	6	123	17.06.2022	1	61
04.06.2020	24	115	02.06.2021	7	4	17.06.2022	2	42
04.06.2020	34	30	02.06.2021	8	50	17.06.2022	3	43
			02.06.2021	9	0	17.06.2022	4	51
			02.06.2021	10	0	17.06.2022	5	12
			02.06.2021	11	2	17.06.2022	6	5
			02.06.2021	12	0	17.06.2022	7	0
			02.06.2021	13	3	17.06.2022	8	7
			02.06.2021	14	6	17.06.2022	9	1
			02.06.2021	15	1	17.06.2022	10	0
			02.06.2021	16	1	17.06.2022	11	0
			03.06.2021	1	77	17.06.2022	12	0
			03.06.2021	2	2	17.06.2022	13	5
			03.06.2021	3	0	17.06.2022	14	5
			03.06.2021	4	0	17.06.2022	15	164
			03.06.2021	5	347	17.06.2022	16	110
			03.06.2021	6	62	17.06.2022	17	0
			03.06.2021	7	4	20.06.2022	1	38
			03.06.2021	8	116	20.06.2022	2	37

03.06.2021	9	1	20.06.2022	3	18
03.06.2021	10	1	20.06.2022	4	159
03.06.2021	11	3	20.06.2022	5	12
03.06.2021	12	1	20.06.2022	6	7
03.06.2021	13	19	20.06.2022	7	0
03.06.2021	14	21	20.06.2022	8	6
03.06.2021	15	24	20.06.2022	9	0
03.06.2021	16	0	20.06.2022	10	0
04.06.2021	1	89	20.06.2022	11	0
04.06.2021	5	414	20.06.2022	12	0
04.06.2021	6	88	20.06.2022	13	0
04.06.2021	8	61	20.06.2022	14	3
15.06.2021	17	0	20.06.2022	15	27
15.06.2021	18	8	20.06.2022	16	165
15.06.2021	19	66	20.06.2022	17	0
15.06.2021	20	2	21.06.2022	1	7
15.06.2021	21	3	21.06.2022	2	6
15.06.2021	22	2	21.06.2022	3	6
15.06.2021	23	1	21.06.2022	4	72
15.06.2021	24	18	21.06.2022	5	1
15.06.2021	25	2	21.06.2022	6	6
15.06.2021	26	42	21.06.2022	7	158
15.06.2021	27	9	21.06.2022	8	111
15.06.2021	28	0	22.06.2022	9	14
15.06.2021	29	11	22.06.2022	10	10
15.06.2021	30	8	22.06.2022	11	2
15.06.2021	31	3	22.06.2022	12	31

15.06.2021	32	0
16.06.2021	17	0
16.06.2021	18	18
16.06.2021	19	34
16.06.2021	20	1
16.06.2021	21	0
16.06.2021	22	4
16.06.2021	23	0
16.06.2021	24	119
16.06.2021	25	2
16.06.2021	26	63
16.06.2021	27	120
16.06.2021	28	2
16.06.2021	29	8
16.06.2021	30	4
16.06.2021	31	4
16.06.2021	32	7
17.06.2021	17	0
17.06.2021	18	2
17.06.2021	19	28
17.06.2021	20	0
17.06.2021	21	0
17.06.2021	22	0
17.06.2021	23	0
17.06.2021	24	16
17.06.2021	25	1
17.06.2021	26	23

22.06.2022	13	3
22.06.2022	14	1
22.06.2022	15	23
22.06.2022	16	5
23.06.2022	1	13
23.06.2022	2	2
23.06.2022	3	0
23.06.2022	4	150
23.06.2022	5	6
23.06.2022	6	2
23.06.2022	15	41
23.06.2022	16	37
23.06.2022	18	0
23.06.2022	19	21

17.06.2021	27	164
17.06.2021	28	5
17.06.2021	29	7
17.06.2021	30	17
17.06.2021	31	5
17.06.2021	32	73
18.06.2021	17	0
18.06.2021	18	37
18.06.2021	19	42
18.06.2021	20	0
18.06.2021	21	0
18.06.2021	22	0
18.06.2021	23	0
18.06.2021	24	12
18.06.2021	25	3
18.06.2021	26	26
18.06.2021	27	217
18.06.2021	28	9
18.06.2021	29	6
18.06.2021	30	60
18.06.2021	31	16
18.06.2021	32	30
21.06.2021	17	0
21.06.2021	18	22
21.06.2021	19	159
21.06.2021	20	0
21.06.2021	21	0

21.06.2021	22	7
21.06.2021	23	1
21.06.2021	24	72
21.06.2021	25	24
21.06.2021	26	24
21.06.2021	27	6
21.06.2021	28	33
21.06.2021	29	7
21.06.2021	30	53
21.06.2021	31	26
21.06.2021	32	250
23.06.2021	33	0
23.06.2021	34	0
23.06.2021	35	0
23.06.2021	36	0
23.06.2021	37	0
23.06.2021	38	0
23.06.2021	39	1
23.06.2021	40	6
23.06.2021	41	0
23.06.2021	42	0
23.06.2021	43	0
23.06.2021	44	0
23.06.2021	45	0
23.06.2021	46	0
23.06.2021	47	1
23.06.2021	48	0

66

Table S2. Quantitative PCR data for comparing the total bacterial community and *Candidatus* Halichondribacter symbioticus between individual sponges.

Adult

**Sponge**	**Larvae**	**Total 16S rDNA**	**Total 16S rDNA (1 ng)**
1	338	106952855.3	21390571.06
10	217	422138056.0	84427611.2
12	147	434801127.6	86960225.52
15	139	287591388.5	57518277.7
19	289	26767180.6	5353436.12
22	139	231021060.2	46204212.04
23	226	179494426.8	35898885.36
24	404	140569375.9	28113875.18
27	157	52056569.8	10411313.96
34	114	59011411.0	11802282.2

Larvae

**Sponge**	**Larvae**	**Total *Ca.* H. symbioticus**	**Total *Ca.* H. symbioticus (1 ng)**
1	338	8165989.9	1633197.98
10	217	6358956.2	1271791.24
12	147	7600807	1520161.4
15	139	7643037.8	1528607.56
19	289	3597532	719506.4
22	139	7357902.9	1471580.58
23	226	5592237.7	1118447.54
24	404	1163314.5	232662.9
27	157	6029171	1205834.2
34	114	9501676.9	1900335.38

Table S3. Quantitative PCR data for comparing estimate host cells (*i.e.*, 18S rDNA).

Sponge	Adult	Larvae
1	2512.6	1067.3
19	2631.5	938.4
23	1986.8	209.5
24	1935.5	799.8
27	2084.7	269.2

Table S4. Quantitative PCR data for individual sponges before and after spawning.

Total bacterial community

Sponge	Pre-spawning	Post-spawning
1	1890540.6	591662.1
12	2842729.4	524867.0
15	2366503.6	648629.5
23	4734063.4	765286.5
27	4238172.5	1407605.5
31	5636869.1	1034767.3
34	5202579.4	936296.7

***Candidatus* Halichondribacter symbioticus**

Sponge	Pre-spawning	Post-spawning
1	16942.1	911.8
12	21614.6	1088.1
15	21247.4	6931.9
23	20908.3	4117.3
27	12443.7	4948.3
31	15549.8	4040.6
34	28379.1	5894.1

Table S5. Quantitative PCR data for adult sponges following antibiotic treatment and juvenile frequency.

Total bacterial community

	**24 h**		**48 h**		
**Replicate**	**Control**	**Antibiotics**	**Control**	**Antibiotics**	**Juvenile (%)**
1	10000.0	346.8	3244.1	322.0	27.7
2	10146.1	1228.3	44514.3	467.4	10.9
3	1132.1	1231.1	12752.6	344.1	11.9
4	5043.1	1098.0	1513.2	673.3	16.3

***Candidatus* Halichondribacter symbioticus**

	**24 h**		**48 h**		
**Replicate**	**Control**	**Antibiotics**	**Control**	**Antibiotics**	**Juvenile (%)**
1	2871.4	2165.7	1501.4	4015.1	6.4
2	1436.8	4199.5	3292.3	3745.4	9.8
3	2350.7	5731.9	5313.7	2839.9	5.6
4	2884.0	6068.8	2458.1	1745.8	10.0
